# Supplementary material for: Topological magnetoplasmon
Source: Nat Commun. 2016 Nov 28;7:13486. doi: 10.1038/ncomms13486 (PMC5148233; doi:10.1038/ncomms13486)
Supplement: Supplementary Information — Supplementary Notes 1-4 and Supplementary References [file ncomms13486-s1.pdf]

Supplementary Note 1: Field-current relation

We give our detailed derivation on the field-current relation Eq. (3) in the main article, starting from the general situation enclosing photon retardation. For a layered system that has continuous translational invariance in the xy-plane, the solutions of Maxwell's equations consist of the basic plane-waves $\mathbf{E} \sim \mathcal{E}\mathrm{e}^{+\mathrm{i}q_x x+\mathrm{i}q_y y+\mathrm{i}\gamma z-\mathrm{i}\omega t}$, $\mathbf{H} \sim \mathcal{H}\mathrm{e}^{+\mathrm{i}q_x x+\mathrm{i}q_y y+\mathrm{i}\gamma z-\mathrm{i}\omega t}$, which can be classified [1] into the H_z-waves ($E_z = 0$),

$$\mathcal{E}_x = +\frac{q_y}{q}\mathcal{C}, \quad \mathcal{E}_y = -\frac{q_x}{q}\mathcal{C}, \quad \mathcal{E}_z = 0, \tag{1}$$

$$\mathcal{H}_x = \frac{c\gamma}{\omega}\frac{q_x}{q}\mathcal{C}, \quad \mathcal{H}_y = \frac{c\gamma}{\omega}\frac{q_y}{q}\mathcal{C}, \quad \mathcal{H}_z = -\frac{cq}{\omega}\mathcal{C}, \tag{2}$$

and E_z-waves ($H_z = 0$),

$$\mathcal{H}_x = -\frac{q_y}{q}\mathcal{D}, \quad \mathcal{H}_y = +\frac{q_x}{q}\mathcal{D}, \quad \mathcal{H}_z = 0, \tag{3}$$

$$\mathcal{E}_x = \frac{c\gamma}{\epsilon\omega}\frac{q_x}{q}\mathcal{D}, \quad \mathcal{E}_y = \frac{c\gamma}{\epsilon\omega}\frac{q_y}{q}\mathcal{D}, \quad \mathcal{E}_z = -\frac{cq}{\epsilon\omega}\mathcal{D}. \tag{4}$$

Here $\mathcal{C}$ and $\mathcal{D}$ are arbitrary constants, ϵ is the dielectric constant in a certain layer (in our model $\epsilon = \epsilon_{\mathrm{A}}$ or ϵ_{B}), and $\gamma = \sqrt{\epsilon\omega^2/c^2 - q^2}$. Note that this classification is consistent with the more familiar transverse-electric (TE) and transverse-magnetic (TM) separation with respect to the in-plane wavenumber q_x, assuming $q_y = 0$ [1, 2].

In our model, the general solution in the upper region $0 < z < +d_{\mathrm{A}}$ of the dielectric constant ϵ_{A} is (omitting the common factor $\mathrm{e}^{+\mathrm{i}q_x x+\mathrm{i}q_y y-\mathrm{i}\omega t}$)

$$\begin{aligned}\mathbf{E}^{>} = &\left\{+\mathbf{e}_x\left[+\frac{q_y}{q}\mathcal{C}_+^{>} + \frac{c\gamma_{\mathrm{A}}}{\epsilon_{\mathrm{A}}\omega}\frac{q_x}{q}\mathcal{D}_+^{>}\right] + \mathbf{e}_y\left[-\frac{q_x}{q}\mathcal{C}_+^{>} + \frac{c\gamma_{\mathrm{A}}}{\epsilon_{\mathrm{A}}\omega}\frac{q_y}{q}\mathcal{D}_+^{>}\right] - \mathbf{e}_z\frac{cq}{\epsilon_{\mathrm{A}}\omega}\mathcal{D}_+^{>}\right\}\mathrm{e}^{+\mathrm{i}\gamma_{\mathrm{A}}z}\\ &+\left\{+\mathbf{e}_x\left[+\frac{q_y}{q}\mathcal{C}_-^{>} - \frac{c\gamma_{\mathrm{A}}}{\epsilon_{\mathrm{A}}\omega}\frac{q_x}{q}\mathcal{D}_-^{>}\right] + \mathbf{e}_y\left[-\frac{q_x}{q}\mathcal{C}_-^{>} - \frac{c\gamma_{\mathrm{A}}}{\epsilon_{\mathrm{A}}\omega}\frac{q_y}{q}\mathcal{D}_-^{>}\right] - \mathbf{e}_z\frac{cq}{\epsilon_{\mathrm{A}}\omega}\mathcal{D}_-^{>}\right\}\mathrm{e}^{-\mathrm{i}\gamma_{\mathrm{A}}z},\end{aligned} \tag{5}$$

$$\begin{aligned}\mathbf{H}^{>} = &\left\{+\mathbf{e}_x\left[-\frac{q_y}{q}\mathcal{D}_+^{>} + \frac{c\gamma_{\mathrm{A}}}{\omega}\frac{q_x}{q}\mathcal{C}_+^{>}\right] + \mathbf{e}_y\left[+\frac{q_x}{q}\mathcal{D}_+^{>} + \frac{c\gamma_{\mathrm{A}}}{\omega}\frac{q_y}{q}\mathcal{C}_+^{>}\right] - \mathbf{e}_z\frac{cq}{\omega}\mathcal{C}_+^{>}\right\}\mathrm{e}^{+\mathrm{i}\gamma_{\mathrm{A}}z}\\ &+\left\{+\mathbf{e}_x\left[-\frac{q_y}{q}\mathcal{D}_-^{>} - \frac{c\gamma_{\mathrm{A}}}{\omega}\frac{q_x}{q}\mathcal{C}_-^{>}\right] + \mathbf{e}_y\left[+\frac{q_x}{q}\mathcal{D}_-^{>} - \frac{c\gamma_{\mathrm{A}}}{\omega}\frac{q_y}{q}\mathcal{C}_-^{>}\right] - \mathbf{e}_z\frac{cq}{\omega}\mathcal{C}_-^{>}\right\}\mathrm{e}^{-\mathrm{i}\gamma_{\mathrm{A}}z}.\end{aligned} \tag{6}$$

Likewise, the general solution in the lower region $-d_{\mathrm{B}} < z < 0$ of the dielectric constant ϵ_{B}

is

$$\mathbf{E}^{<} = \left\{+\mathbf{e}_x\left[+\frac{q_y}{q}\mathcal{C}_+^{<} + \frac{c\gamma_{\rm B}}{\epsilon_{\rm B}\omega}\frac{q_x}{q}\mathcal{D}_+^{<}\right] + \mathbf{e}_y\left[-\frac{q_x}{q}\mathcal{C}_+^{<} + \frac{c\gamma_{\rm B}}{\epsilon_{\rm B}\omega}\frac{q_y}{q}\mathcal{D}_+^{<}\right] - \mathbf{e}_z\frac{cq}{\epsilon_{\rm B}\omega}\mathcal{D}_+^{<}\right\}{\rm e}^{+{\rm i}\gamma_{\rm B}z}$$
$$+ \left\{+\mathbf{e}_x\left[+\frac{q_y}{q}\mathcal{C}_-^{<} - \frac{c\gamma_{\rm B}}{\epsilon_{\rm B}\omega}\frac{q_x}{q}\mathcal{D}_-^{<}\right] + \mathbf{e}_y\left[-\frac{q_x}{q}\mathcal{C}_-^{<} - \frac{c\gamma_{\rm B}}{\epsilon_{\rm B}\omega}\frac{q_y}{q}\mathcal{D}_-^{<}\right] - \mathbf{e}_z\frac{cq}{\epsilon_{\rm B}\omega}\mathcal{D}_-^{<}\right\}{\rm e}^{-{\rm i}\gamma_{\rm B}z}, \quad (7)$$

$$\mathbf{H}^{<} = \left\{+\mathbf{e}_x\left[-\frac{q_y}{q}\mathcal{D}_+^{<} + \frac{c\gamma_{\rm B}}{\omega}\frac{q_x}{q}\mathcal{C}_+^{<}\right] + \mathbf{e}_y\left[+\frac{q_x}{q}\mathcal{D}_+^{<} + \frac{c\gamma_{\rm B}}{\omega}\frac{q_y}{q}\mathcal{C}_+^{<}\right] - \mathbf{e}_z\frac{cq}{\omega}\mathcal{C}_+^{<}\right\}{\rm e}^{+{\rm i}\gamma_{\rm B}z}$$
$$+ \left\{+\mathbf{e}_x\left[-\frac{q_y}{q}\mathcal{D}_-^{<} - \frac{c\gamma_{\rm B}}{\omega}\frac{q_x}{q}\mathcal{C}_-^{<}\right] + \mathbf{e}_y\left[+\frac{q_x}{q}\mathcal{D}_-^{<} - \frac{c\gamma_{\rm B}}{\omega}\frac{q_y}{q}\mathcal{C}_-^{<}\right] - \mathbf{e}_z\frac{cq}{\omega}\mathcal{C}_-^{<}\right\}{\rm e}^{-{\rm i}\gamma_{\rm B}z}. \quad (8)$$

The boundary conditions at the perfect-metal surface $z = +d_{\rm A}$ are

$$E_x^{>}|_{z=+d_{\rm A}} = 0, \quad E_y^{>}|_{z=+d_{\rm A}} = 0, \quad (9)$$

i.e.,

$$+\frac{q_y}{q}\left(\mathcal{C}_+^{>}{\rm e}^{+{\rm i}\gamma_{\rm A}d_{\rm A}} + \mathcal{C}_-^{>}{\rm e}^{-{\rm i}\gamma_{\rm A}d_{\rm A}}\right) + \frac{c\gamma_{\rm A}}{\epsilon_{\rm A}\omega}\frac{q_x}{q}\left(\mathcal{D}_+^{>}{\rm e}^{+{\rm i}\gamma_{\rm A}d_{\rm A}} - \mathcal{D}_-^{>}{\rm e}^{-{\rm i}\gamma_{\rm A}d_{\rm A}}\right) = 0, \quad (10)$$
$$-\frac{q_x}{q}\left(\mathcal{C}_+^{>}{\rm e}^{+{\rm i}\gamma_{\rm A}d_{\rm A}} + \mathcal{C}_-^{>}{\rm e}^{-{\rm i}\gamma_{\rm A}d_{\rm A}}\right) + \frac{c\gamma_{\rm A}}{\epsilon_{\rm A}\omega}\frac{q_y}{q}\left(\mathcal{D}_+^{>}{\rm e}^{+{\rm i}\gamma_{\rm A}d_{\rm A}} - \mathcal{D}_-^{>}{\rm e}^{-{\rm i}\gamma_{\rm A}d_{\rm A}}\right) = 0, \quad (11)$$

and at the perfect-metal surface $z = -d_{\rm B}$ are

$$E_x^{<}|_{z=-d_{\rm B}} = 0, \quad E_y^{<}|_{z=-d_{\rm B}} = 0, \quad (12)$$

i.e.,

$$+\frac{q_y}{q}\left(\mathcal{C}_+^{<}{\rm e}^{-{\rm i}\gamma_{\rm B}d_{\rm B}} + \mathcal{C}_-^{<}{\rm e}^{+{\rm i}\gamma_{\rm B}d_{\rm B}}\right) + \frac{c\gamma_{\rm B}}{\epsilon_{\rm B}\omega}\frac{q_x}{q}\left(\mathcal{D}_+^{<}{\rm e}^{-{\rm i}\gamma_{\rm B}d_{\rm B}} - \mathcal{D}_-^{<}{\rm e}^{+{\rm i}\gamma_{\rm B}d_{\rm B}}\right) = 0, \quad (13)$$
$$-\frac{q_x}{q}\left(\mathcal{C}_+^{<}{\rm e}^{-{\rm i}\gamma_{\rm B}d_{\rm B}} + \mathcal{C}_-^{<}{\rm e}^{+{\rm i}\gamma_{\rm B}d_{\rm B}}\right) + \frac{c\gamma_{\rm B}}{\epsilon_{\rm B}\omega}\frac{q_y}{q}\left(\mathcal{D}_+^{<}{\rm e}^{-{\rm i}\gamma_{\rm B}d_{\rm B}} - \mathcal{D}_-^{<}{\rm e}^{+{\rm i}\gamma_{\rm B}d_{\rm B}}\right) = 0. \quad (14)$$

The boundary conditions at the $z = 0$ plane are

$$E_x^{>}|_{z=0} = E_x|_{z=0} = E_x^{<}|_{z=0}, \quad E_y^{>}|_{z=0} = E_y|_{z=0} = E_y^{<}|_{z=0}, \quad (15)$$

and

$$\mathbf{e}_z \times \{\mathbf{H}^{>}|_{z=0} - \mathbf{H}^{<}|_{z=0}\} = \frac{4\pi}{c}\mathbf{j}. \quad (16)$$

I.e.,

$$+\frac{q_y}{q}\left(\mathcal{C}_+^{>} + \mathcal{C}_-^{>}\right) + \frac{c\gamma_{\rm A}}{\epsilon_{\rm A}\omega}\frac{q_x}{q}\left(\mathcal{D}_+^{>} - \mathcal{D}_-^{>}\right) = E_x|_{z=0} = +\frac{q_y}{q}\left(\mathcal{C}_+^{<} + \mathcal{C}_-^{<}\right) + \frac{c\gamma_{\rm B}}{\epsilon_{\rm B}\omega}\frac{q_x}{q}\left(\mathcal{D}_+^{<} - \mathcal{D}_-^{<}\right), \quad (17)$$

$$-\frac{q_x}{q}\left(\mathcal{C}_+^> + \mathcal{C}_-^>\right) + \frac{c\gamma_{\rm A}}{\epsilon_{\rm A}\omega}\frac{q_y}{q}\left(\mathcal{D}_+^> - \mathcal{D}_-^>\right) = E_y|_{z=0} = -\frac{q_x}{q}\left(\mathcal{C}_+^< + \mathcal{C}_-^<\right) + \frac{c\gamma_{\rm B}}{\epsilon_{\rm B}\omega}\frac{q_y}{q}\left(\mathcal{D}_+^< - \mathcal{D}_-^<\right), \tag{18}$$

and

$$-\frac{q_y}{q}\left[\mathcal{D}_+^> + \mathcal{D}_-^> - \mathcal{D}_+^< - \mathcal{D}_-^<\right] + \frac{c}{\omega}\frac{q_x}{q}\left[\gamma_{\rm A}\mathcal{C}_+^> - \gamma_{\rm A}\mathcal{C}_-^> - \gamma_{\rm B}\mathcal{C}_+^< + \gamma_{\rm B}\mathcal{C}_-^<\right] = \frac{4\pi}{c}j_y, \tag{19}$$

$$-\frac{q_x}{q}\left[\mathcal{D}_+^> + \mathcal{D}_-^> - \mathcal{D}_+^< - \mathcal{D}_-^<\right] - \frac{c}{\omega}\frac{q_y}{q}\left[\gamma_{\rm A}\mathcal{C}_+^> - \gamma_{\rm A}\mathcal{C}_-^> - \gamma_{\rm B}\mathcal{C}_+^< + \gamma_{\rm B}\mathcal{C}_-^<\right] = \frac{4\pi}{c}j_x, \tag{20}$$

where $\mathbf{j}$ is the 2D current density in the 2DEG at $z = 0$.

Solving the above linear equations altogether, we get

$$\mathcal{C}_+^> = \frac{+q_y E_x|_{z=0} - q_x E_y|_{z=0}}{q(1 - {\rm e}^{+2{\rm i}\gamma_{\rm A} d_{\rm A}})}, \tag{21}$$

$$\mathcal{C}_-^> = \frac{+q_y E_x|_{z=0} - q_x E_y|_{z=0}}{q(1 - {\rm e}^{-2{\rm i}\gamma_{\rm A} d_{\rm A}})}, \tag{22}$$

$$\mathcal{D}_+^> = +\frac{\omega\epsilon_{\rm A}}{c\gamma_{\rm A}}\frac{q_x E_x|_{z=0} + q_y E_y|_{z=0}}{q(1 - {\rm e}^{+2{\rm i}\gamma_{\rm A} d_{\rm A}})}, \tag{23}$$

$$\mathcal{D}_-^> = -\frac{\omega\epsilon_{\rm A}}{c\gamma_{\rm A}}\frac{q_x E_x|_{z=0} + q_y E_y|_{z=0}}{q(1 - {\rm e}^{-2{\rm i}\gamma_{\rm A} d_{\rm A}})}, \tag{24}$$

and

$$\mathcal{C}_+^< = \frac{+q_y E_x|_{z=0} - q_x E_y|_{z=0}}{q(1 - {\rm e}^{-2{\rm i}\gamma_{\rm B} d_{\rm B}})}, \tag{25}$$

$$\mathcal{C}_-^< = \frac{+q_y E_x|_{z=0} - q_x E_y|_{z=0}}{q(1 - {\rm e}^{+2{\rm i}\gamma_{\rm B} d_{\rm B}})}, \tag{26}$$

$$\mathcal{D}_+^< = +\frac{\omega\epsilon_{\rm B}}{c\gamma_{\rm B}}\frac{q_x E_x|_{z=0} + q_y E_y|_{z=0}}{q(1 - {\rm e}^{-2{\rm i}\gamma_{\rm B} d_{\rm B}})}, \tag{27}$$

$$\mathcal{D}_-^< = -\frac{\omega\epsilon_{\rm B}}{c\gamma_{\rm B}}\frac{q_x E_x|_{z=0} + q_y E_y|_{z=0}}{q(1 - {\rm e}^{+2{\rm i}\gamma_{\rm B} d_{\rm B}})}. \tag{28}$$

Finally, we can obtain the relation

$$-\frac{1}{q^2}\begin{pmatrix} q_x^2 f + q_y^2 g & q_x q_y (f - g) \\ q_x q_y (f - g) & q_y^2 f + q_x^2 g \end{pmatrix}\begin{pmatrix} E_x|_{z=0} \\ E_y|_{z=0} \end{pmatrix} = \frac{4\pi}{c}\begin{pmatrix} j_x \\ j_y \end{pmatrix}, \tag{29}$$

where

$$f = {\rm i}\frac{\omega}{c}\left\{\frac{\epsilon_{\rm A}}{\gamma_{\rm A}}\cot(\gamma_{\rm A} d_{\rm A}) + \frac{\epsilon_{\rm B}}{\gamma_{\rm B}}\cot(\gamma_{\rm B} d_{\rm B})\right\}, \tag{30}$$

$$g = {\rm i}\frac{c}{\omega}\left\{\gamma_{\rm A}\cot(\gamma_{\rm A} d_{\rm A}) + \gamma_{\rm B}\cot(\gamma_{\rm B} d_{\rm B})\right\}. \tag{31}$$

A matrix inversion leads to

$$\begin{pmatrix} E_x|_{z=0} \\ E_y|_{z=0} \end{pmatrix} = -\frac{4\pi}{cq^2 fg} \begin{pmatrix} q_x^2 g + q_y^2 f & q_x q_y (g-f) \\ q_x q_y (g-f) & q_y^2 g + q_x^2 f \end{pmatrix} \begin{pmatrix} j_x \\ j_y \end{pmatrix}. \tag{32}$$

In the nonretarded limit $q \gg \omega/c$, $\gamma_{\rm A} \sim \gamma_{\rm B} \sim {\rm i}q$, it gives

$$\begin{aligned} \begin{pmatrix} E_x|_{z=0} \\ E_y|_{z=0} \end{pmatrix} &= \frac{4\pi}{{\rm i}\omega q \left\{\epsilon_{\rm A}\coth(qd_{\rm A}) + \epsilon_{\rm B}\coth(qd_{\rm B})\right\}} \begin{pmatrix} q_x^2 & q_x q_y \\ q_x q_y & q_y^2 \end{pmatrix} \begin{pmatrix} j_x \\ j_y \end{pmatrix} \\ &= \frac{2\pi}{{\rm i}\omega q \xi(q)} \begin{pmatrix} q_x^2 & q_x q_y \\ q_x q_y & q_y^2 \end{pmatrix} \begin{pmatrix} j_x \\ j_y \end{pmatrix}, \end{aligned} \tag{33}$$

where $\xi(q) \equiv \frac{1}{2}\left\{\epsilon_{\rm A}\coth(qd_{\rm A}) + \epsilon_{\rm B}\coth(qd_{\rm B})\right\}$ is a q-dependent screening function, as used in the main article.

Supplementary Note 2: Inclusion of photon retardation

We now consider the influence from photon retardation. An asymmetric structure used above is hard to pursue. For simplicity, let us consider a symmetric structure here by letting $d_{\rm A} = d_{\rm B} = d$, $\epsilon_{\rm A} = \epsilon_{\rm B} = \epsilon$, $\gamma_{\rm A} = \gamma_{\rm B} = \gamma \equiv {\rm i}\beta$, where $\beta \equiv \sqrt{q^2 - \epsilon\omega^2/c^2}$. According to Eq. (29–32), in the retarded case,

$$\begin{pmatrix} E_x|_{z=0} \\ E_y|_{z=0} \end{pmatrix} = \frac{2\pi}{{\rm i}\omega} \frac{\tanh(\beta d)}{\beta\epsilon} \begin{pmatrix} q_x^2 - \epsilon\dfrac{\omega^2}{c^2} & q_x q_y \\ q_x q_y & q_y^2 - \epsilon\dfrac{\omega^2}{c^2} \end{pmatrix} \begin{pmatrix} j_x \\ j_y \end{pmatrix}, \tag{34}$$

and in the nonretarded case,

$$\begin{pmatrix} E_x|_{z=0} \\ E_y|_{z=0} \end{pmatrix} = \frac{2\pi}{{\rm i}\omega} \frac{\tanh(qd)}{q\epsilon} \begin{pmatrix} q_x^2 & q_x q_y \\ q_x q_y & q_y^2 \end{pmatrix} \begin{pmatrix} j_x \\ j_y \end{pmatrix}. \tag{35}$$

We can again investigate the bulk modes by assuming uniform equilibrium density and magnetic field. Combining Eq. (2) in the main article and Eq. (34) here (in place of Eq. (3) in the main article), we can obtain a nonlinear eigenvalue equation,

$$\omega^2 - \omega_{\rm c}^2 + \left(2\eta\omega^2 - v_{\rm p}^2 q^2\right)\frac{\tanh(\beta d)}{\beta d} + \eta\left(\eta\omega^2 - v_{\rm p}^2 q^2\right)\frac{\tanh^2(\beta d)}{(\beta d)^2} = 0. \tag{36}$$

Here we have removed the $\omega = 0$ solutions and have defined a dimensionless factor $\eta = \epsilon v_{\rm p}^2/c^2 = 2\pi e^2 n_0 d/m_* c^2$. It is the squared ratio between $v_{\rm p}$ and $(c/\sqrt{\epsilon})$; the latter is just the light speed in the surrounding dielectric. One can plot the new bulk spectrum

using Eq. (36), and shall find that the MP spectrum becomes slightly bent towards the frequency zero but is still gapped.

Note that our model system is a parallel-plate metal waveguide. The portion of MP modes lying inside the light cone with a gap $\omega_{\rm c}$ can couple with the waveguide modes with a gap $\omega_{\rm min} = c\pi/d\sqrt{\epsilon}$, commonly known as the cutoff frequency [2]. However, MP modes do not couple with the gapless transverse-electromagnetic (TEM) mode, because the electric field of TEM modes is completely along the z-direction [2] and so cannot induce the electron motion restricted in the xy-plane. Since the coupling of gapped MP modes with the gapped waveguide modes (regardless of how large or how small the gaps are) does not involve a band closing and reopening process at the frequency zero, one can immediately deduce that such a coupling will not cause a topological phase transition. Therefore, our topological argument in the main article in the nonretarded formalism is robust against the inclusion of photon retardation.

Supplementary Note 3: Comparison to ordinary surface plasmon

We point out that our results do not apply for the ordinary surface plasmon (SP) on the surface of a 3D metal. SP by itself is gapped at a finite frequency $\omega_{\rm sp}$ [3, 4] due to the charge screening from the semi-infinite bulk metal. The apparent "gap closing" of surface plasmon polariton (SPP) is caused by the retarded coupling to photons rather than any intrinsic electronic behaviors. This is in stark contrast to the initially gapless 2D plasmon here, which then experiences a topological "gap opening" under a magnetic field.

Supplementary Note 4: Zero-frequency solutions for a hollow disk geometry

In the polar coordinates, and let $j_{\rm D} \equiv v_{\rm p}\varrho$, the long-wavelength equations of motion are

$$-\mathrm{i}\omega j_{\rm D} = -v_{\rm p}\partial_r j_r - v_{\rm p}\frac{1}{r}j_r - v_{\rm p}\frac{1}{r}\partial_\phi j_\phi, \tag{37}$$

$$-\mathrm{i}\omega j_r = -v_{\rm p}\partial_r j_{\rm D} - \omega_{\rm c} j_\phi, \tag{38}$$

$$-\mathrm{i}\omega j_\phi = -v_{\rm p}\frac{1}{r}\partial_\phi j_{\rm D} + \omega_{\rm c} j_r. \tag{39}$$

Taking account of the cylindrical symmetry $\sim \mathrm{e}^{\mathrm{i}\nu\phi}$, and seeking the $\omega = 0$ solutions only, we

have

$$0 = \partial_r j_r(r) + \frac{1}{r} j_r(r) + \frac{\mathrm{i}\nu}{r} j_\phi(r), \tag{40}$$

$$j_\phi(r) = -\frac{v_\mathrm{p}}{\omega_\mathrm{c}} \partial_r j_\mathrm{D}(r), \tag{41}$$

$$j_r(r) = \frac{v_\mathrm{p}}{\omega_\mathrm{c}} \frac{\mathrm{i}\nu}{r} j_\mathrm{D}(r). \tag{42}$$

For an arbitrarily given $j_\mathrm{D}(r)$, Eqs. (41) and (42) generate $j_\phi(r)$ and $j_r(r)$, which automatically satisfy Eq. (40). Therefore, Eq. (40) can be removed.

Let us now consider a system with a hole of radius a in the center. There come two distinct classes of solutions:

1. $\nu = 0$. Then $j_r(r) = 0$ forever holds. The condition of the no-normal-current at the boundary $r = a$ is always satisfied. Then for a whatever given $j_\mathrm{D}(r)$ profile, we just have a derived $j_\phi(r) = -\frac{v_\mathrm{p}}{\omega_\mathrm{c}} \partial_r j_\mathrm{D}(r)$.

2. $\nu \neq 0$. Then the condition of the no-normal-current at the boundary $r = a$ enforces $j_\mathrm{D}(a) = 0$. Any $j_\mathrm{D}(r)$ satisfying this requirement is allowed. And we have a derived $j_\phi(r) = -\frac{v_\mathrm{p}}{\omega_\mathrm{c}} \partial_r j_\mathrm{D}(r)$, and derived $j_r(r) = \frac{v_\mathrm{p}}{\omega_\mathrm{c}} \frac{\mathrm{i}\nu}{r} j_\mathrm{D}(r)$.

All the $\nu \neq 0$ solutions are obviously bulk solutions within the zero-frequency band. $j_\mathrm{D}(a) = 0$ and $j_r(a) = 0$ completely disconnects the 2DEG with the "outside world". All the $\nu = 0$ solutions are obviously orthogonal to the $\nu \neq 0$ solutions under angular integration. They can be edge-like or bulk like.

The zero-mode edge solution given in the main article after taking $\omega \to 0$ from the finite frequencies is only one of the many allowed zero-frequency solutions here. Hence it cannot be physically isolated from the highly degenerate zero-frequency band here. This is one of the most extraordinary properties of this 3-band topological system.

---

[1] Chew, W. C. *Waves and Fields in Inhomogeneous Media* (IEEE Press, New York, 1995).

[2] Jackson, J. D. *Classical Electrodynamics* (Wiley, 1998), 3rd edn.

[3] Economou, E. N. Surface plasmons in thin films. *Phys. Rev.* **182**, 539 (1969).

[4] Liebsch, A. *Electronic excitations at metal surfaces* (Springer Science & Business Media, 2013).
